# Structure–Function Analysis of the Steroid-Hydroxylating Cytochrome P450 109 (CYP109) Enzyme Family

**DOI:** 10.3390/ijms26136219

**Published:** 2025-06-27

**Authors:** Siphesihle M. Msweli, Tiara Padayachee, Thembeka Khumalo, David R. Nelson, David C. Lamb, Khajamohiddin Syed

**Affiliations:** 1 Department of Biochemistry and Microbiology, Faculty of Science, Agriculture and Engineering, University of Zululand, Empangeni 3886, South Africa; mooisphesihle@gmail.com (S.M.M.); teez07padayachee@gmail.com (T.P.); tirikhumalo@gmail.com (T.K.); 2 Department of Microbiology, Immunology and Biochemistry, University of Tennessee Health Science Center, Memphis, TN 38163, USA; drnelson1@gmail.com; 3 Faculty of Medicine, Health and Life Sciences, Swansea University, Swansea SA2 8PP, UK

**Keywords:** cytochrome P450, CYP109, steroid, oxidation reactions, active site, crystal structure

## Abstract

Steroids are found in bacteria and eukaryotes, and genes potentially encoding steroid metabolic enzymes have also been identified in giant viruses. For decades, hydroxylated steroids have been utilized in medicine to treat various human diseases. The hydroxylation of steroids can be achieved using microbial enzymes, especially cytochrome P450 monooxygenases (CYPs/P450s) and is well documented. Understanding the structural determinants that govern the regio- and stereoselectivity of steroid hydroxylation by P450s is essential in order to fully exploit their potential. Herein, we present a comprehensive analysis of the steroid-hydroxylating CYP109 family across the domains of life and delineate the structural determinants that govern steroid hydroxylation. Data mining, annotation, and phylogenetic analysis revealed that CYP109 family members are highly populated in bacteria, and indeed, these members passed from bacteria to archaea by horizontal gene transfer, leading to the evolution of P450s in archaea. Analysis of twelve CYP109 crystal structures revealed large, flexible, and dynamic active site cavities that can accommodate multiple ligands. The correct positioning and orientation of the steroid in the active site cavity and the nature of the C17 substituent on the steroid molecule influence catalysis. In an analogous fashion to the CYP107 family, the amino acid residues within the CYP109 binding pocket involve hydrophilic and hydrophobic interactions, influencing substrate orientations and anchoring and determining the site of hydroxylation and catalytic activity. A handful of amino acids, such as Val84, Val292, and Ser387 in CYP109B4, have been found to play a role in determining the catalytic regiospecificity, and a single amino acid, such as Arg74 in CYP109A2, has been found to be essential for the enzymatic activity. This work serves as a reference for the precise understanding of CYP109 structure–function relationships and for P450 enzymes in general. The findings will guide the genetic engineering of CYP109 enzymes to produce valuable steroid molecules of medicinal and biotechnological importance.

## 1. Introduction

Steroids are organic molecules produced by organisms across the domains of life. These compounds perform essential biological functions, such as controlling cell membrane fluidity and acting as signaling molecules. Since their identification 90 years ago, their applications in the treatment of many diseases have become well established [1,2,3]. The main functions of steroids include acting as anti-inflammatory or immune-modulatory agents. Steroids remain one of the most valuable and widely used class of drugs.

Hydroxylated steroids exert higher biological activity compared with their non-hydroxylated parent compounds [4,5,6]. For this reason, the generation of hydroxylated steroids is always in demand. Enzymatic hydroxylation of steroids is a favorable route toward hydroxylated steroids compared with synthetic chemistry methods, as regio- and stereoselective positioning of hydroxy groups at nonactivated sites is a big challenge. The synthesis of steroids using chemical methods more often involves multi-step processes with overall low production yield, and they are environmentally unfriendly processes.

The microbial conversion of steroids into different hydroxylated products, especially using different enzymes, is gaining momentum [7,8]. Among the enzymes utilized for the generation of hydroxylated steroids, cytochrome P450 monooxygenases (CYPs/P450s) are highly sought after, as they perform exquisite regio- and stereospecific hydroxylation of inert C-H bonds [6]. P450s are heme-containing enzymes ubiquitously found in all the domains of life, including viruses [9]. P450s have been classified into families and subfamilies based on their amino acid identity, with members of the same family sharing more than 40% sequence identity and those belonging to the same subfamily sharing more than 55% sequence identity [10].

**Figure 1:**
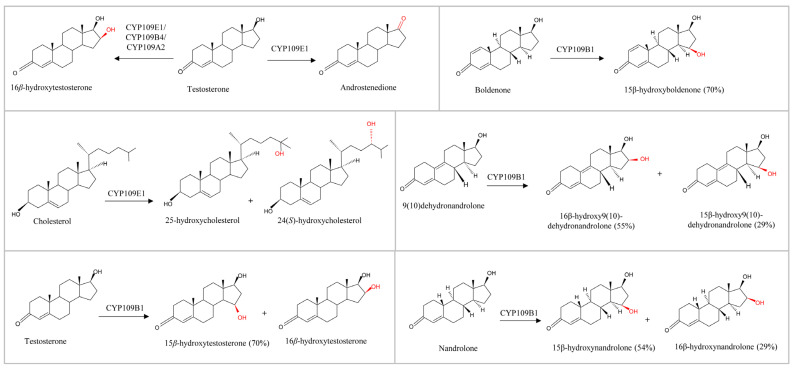
Schematic diagram illustrating examples of CYP109 enzymes’ hydroxylation of various steroids. CYP109s introduction of hydroxyl groups are indicated in red. The percentage of product yield is shown in parentheses next to its name.

Specific P450s with steroid hydroxylation capability have been reported in the literature [7,8]. Among these P450s, the CYP109 family members are known to perform regio- and stereospecific oxidation of various steroids and other molecules (Figure 1). The CYP109 family members have been found in various bacterial species such as those belonging to the genera *Bacillus* and *Myxobacteria* [11,12]. Some CYP109 members have been well characterized biochemically, and their functions are well known. Here we provide some of the CYP109 members’ functions.

CYP109E1 from *Bacillus megaterium* catalyzes the oxidation of testosterone into 16*β*-hydroxytestosterone (major product) and androstenedione (minor product) in a regio- and stereospecific manner (Figure 1) [13]. CYP109E1 oxidizes cholesterol in a selective manner, producing the oxysterols 24(*S*)-hydroxycholesterol and 25-hydroxycholesterol (Figure 1) [14]. CYP109E1 can also catalyze the hydroxylation of vitamin D3 (VD3) at carbons C-24 and C-25, resulting in the formation of 24(*S*)-hydroxyvitamin D3 (24*S*(OH)VD3), 25-hydroxyvitamin D3(25(OH)VD3), and 24*S*,25-dihydroxyvitamin D3(24*S*,25(OH)2VD3) [15]. 24*S*(OH)VD3 was found to be a CYP109E1 intermediate product before its final conversion to 24*S*,25(OH)2VD3 [15]. The CYP109E1 enzyme can also hydroxylate vitamin D2, resulting in the formation of 25-OHVD2, which is further converted into 24(R),25-diOHVD2 [16].

CYP109A2 from *B. megaterium*, in a similar fashion to CYP109E1, oxidizes testosterone in a highly regio- and stereoselective manner to 16*β*-hydroxytestosterone (Figure 1) [17] and also hydroxylates vitamin D3 to 25-hydroxyvitamin D3 [18]. CYP109B4 from *Bacillus sonorensis* oxidizes testosterone to 16*β*-hydroxytestosterone (Figure 1) [19]. A detailed functional characterization of CYP109B1 from *Bacillus subtilis* has been conducted, elucidating its regio- and stereoselectivity toward various steroids. CYP109B1 oxidizes testosterone and boldenone to produce their 15*β*-hydroxylated forms with a 70% product yield. In the case of nandrolone, two oxidized products are formed: the 15*β*-hydroxylated nandrolone is the primary product, while the 16*β*-hydroxylated nandrolone is produced in smaller amounts (29%) (Figure 1) [20]. However, in the case of 9(10)dehydronandrolone, the 16*β*-hydroxylated product is the primary product (55%), and the 15*β*-hydroxylated product is the minor product (29%) (Figure 1) [20]. Furthermore, CYP109B1 oxidizes valencene to nootkatol and nootkatone, compounds that are used in perfumes, food flavoring, and as insecticides, and it converts compactin to pravastatin, important classes of cholesterol-lowering agents (statins) [21].

CYP109C2 and CYP109D1 enzymes from *Sorangium cellulosum* So ce56 have been shown to hydroxylate saturated fatty acids, including lauric acid, myristic acid, tridecanoic acid, palmitic acid, and capric acid [11]. Both CYP109C2 and CYP109D1, as well as CYP109B1 from *B. subtilis,* oxidize α-ionone into 3OH-α-ionone and β-ionone into 4-OH-*β*-ionone, used as fragrances in perfumes and cosmetics [21,22].

Recently, it has been proposed that CYP109 genes, along with CYP147 and CYP197 P450 genes, have been transferred from bacteria to archaea [12]. Subsequently, these P450 families led to the evolution of P450s in archaea [12]. Only a handful of CYP109 members from bacteria have been analyzed as part of the study, and the complete distribution of CYP109 members in bacteria still needs to be elucidated.

Despite the medical and biotechnological importance of CYP109 members in the generation of hydroxylated steroids, a detailed investigation of CYP109 protein structures in terms of active site cavity molecular dynamics and the key amino acids interacting with bound ligands has yet to be undertaken. Furthermore, genome-wide analysis of CYP109 genes will give a more complete understanding of this family with respect to its evolution in archaea. The present study addresses these two research knowledge gaps by performing genome-wide analysis of CYP109 members across the domains of life and identifies the molecular details governing the CYP109 regio- and stereoselectivity of these steroids.

## 2. Results

### 2.1. CYP109s Are Present Only in Bacteria and Archaea to Date

**Figure 2:**
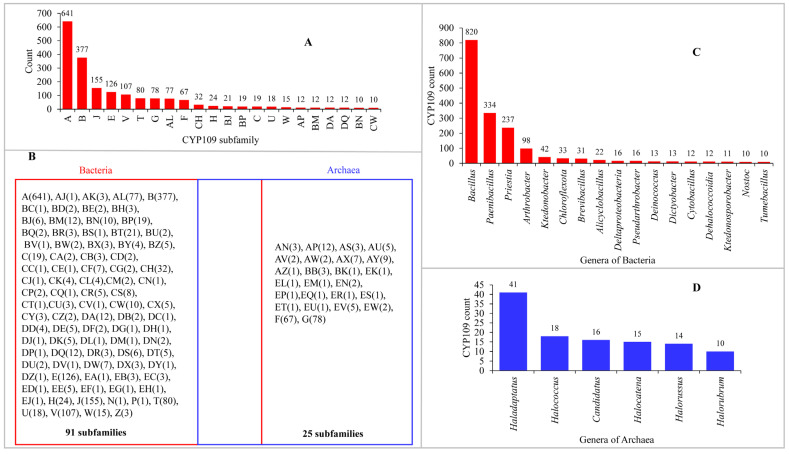
Analysis of CYP109 genes in bacteria and archaea. (**A**) Comparative analysis of CYP109 subfamily members. (**B**) Comparative analysis of CYP109 subfamilies between bacteria and archaea. The number in brackets denotes the number of members in that subfamily. (**C**) Comparative analysis of CYP109s among bacterial genera. (**D**) Comparative analysis of CYP109s among archaeal genera. In panels (**A**,**C**,**D**), the numbers next to bars indicate the number of members for a particular subfamily (for (**A**)) and in the particular genera (in (**C**,**D**)). Only members with more than 10 for a subfamily or genus are shown in panels (**A**,**C**,**D**). Detailed information is presented in Tables S2 and S3.

Data mining and annotation (assigning P450 family and subfamilies) resulted in the identification of 2158 CYP109s at the NCBI (https://www.ncbi.nlm.nih.gov/gene/; accessed on 25 April 2025) from the initial 5000 hits (Table S1). Only species belonging to bacteria and archaea encode CYP109s to date (Table S1). Bacteria encode the majority of CYP109 genes, possessing 1947 CYP109s genes compared with 211 CYP109s encoded in archaea (Table S1). The 2158 CYP109s are grouped into 116 subfamilies, of which 22 subfamilies have more than 10 members (Figure 2A and Table S2). Among the CYP109s, subfamily A has the highest number of members (641), followed by subfamily B (377), subfamily J (155), subfamily E (126), and subfamily V (107) (Figure 2A and Table S2). A comparative analysis of the subfamilies revealed that bacterial species have the highest number of CYP109 subfamilies (91) compared with archaeal species, which have 25 (Figure 2B). Interestingly, none of the CYP109 subfamilies are common between bacteria or archaea (Figure 2B), indicating the divergence of CYP109 genes after the lateral gene transfer (LGT). Among the bacterial CYP109 subfamilies, subfamily A has the highest number of members (641), followed by subfamily B with 377 members, subfamily J with 155 members, subfamily E with 126 members, and subfamily V with 107 members (Figure 2B and Table S2). Among the archaeal CYP109 subfamilies, subfamily G has the most members (78), followed by subfamilies F and AP with 67 and 12, respectively (Figure 2B and Table S2). These results indicate that the CYP109 subfamilies are enriched or bloomed (the presence of many copies of the same family or subfamily of genes in a species) in bacteria and archaea. The blooming of certain P450 families or subfamilies within a family has been attributed to the adaptation of a species to its ecological niches or toward its hosts [23]. It has also been argued that blooms are a stochastic process that matches a power law and is not driven by selection [24].

Analysis of the CYP109 distribution patterns revealed that these P450s are present in species of 98 bacterial genera and 43 archaeal genera (Figure 2C,D and Table S3). Among these genera, only 17 from bacteria and 6 from archaea have more than 10 CYP109 genes (Figure 2C,D), indicating that these P450s are highly populated in specific genera. Among the bacterial genera, *Bacillus* had the highest number of CYP109s (820), followed by *Paenibacillus* with 334 CYP109s and *Priestia* with 237 CYP109s (Figure 2C and Table S3). Among archaeal genera, *Haladaptatus* has the highest number of CYP109s (41), followed by *Halococcus* with 18 CYP109s and *Candidatus* with 16 CYP109s (Figure 2D and Table S3).

### 2.2. CYP109s Are of Bacterial Origin

**Figure 3:**
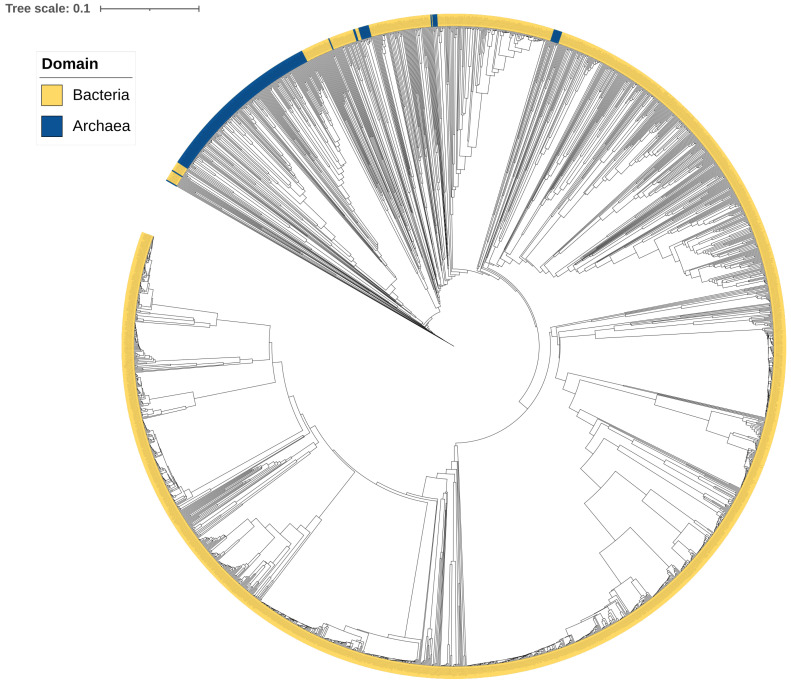
Phylogenetic analysis of CYP109 family members. CYP109 family members belonging to bacteria and archaea are displayed in different colors. A high-resolution phylogenetic tree in PDF format is provided in the (Supplementary Material Figure S1), where P450s on each branch can be discerned.

In a previous study, we proposed that archaeal P450s are bacterial in origin, following horizontal gene transfer, and probably diverged from CYP109, CYP147, and CYP197 genes [12]. The results of the present study, in which we performed a comprehensive genomic analysis of CYP109s, further supported this hypothesis. This is consistent with previous research results, which show that bacteria have more CYP109 gene sequences (1947 vs. 211 P450s) and have the highest CYP109 subfamily diversity (91 vs. 25 subfamilies) compared with archaea (Figure 2). These results, along with the fact that CYP109s are also encoded in archaeal plasmids and chromosomal DNA [12], indicate CYP109 lateral gene transfer from bacteria to archaea. Phylogenetic analysis of CYP109s indicated both possible protein structural conservation and species-specific changes occurring in CYP109s. The majority of the archaeal CYP109s are grouped together, although some are scattered on the tree, indicating their evolutionary linkage with bacterial CYP109s (Figure 3).

### 2.3. CYP109 Members Are Structurally Highly Dynamic

Twelve CYP109 crystal structures are available at RCSB PDB [25] (Table 1). Among these structures, seven are in the closed conformation (bound with ligand and heme), and five of them are in the open conformation (containing only the heme cofactor) (Table 1). The surface area of the active site cavity of the CYP109 proteins in open conformation ranges between 1160 Å^2^ (CYP109Q5) and 1682 Å^2^ (CYP109B1), whereas the surface area of the active site cavity in closed conformation ranges between 806 Å^2^ (CYP109A2) and 2021 Å^2^ (CYP109E1) (Table 1). The volume of the active site in an open conformation ranges between 769 Å^3^ (CYP109Q5) and 2699 Å^3^ (CYP109E1) compared with 446 Å^3^ (CYP109A2) and 2809 Å^3^ (CYP109E1) in a closed conformation (Table 1). This data indicates that CYP109s are highly flexible and can accommodate a range of small to large substrates, the same as observed for CYP107 P450s [26].

An interesting pattern was observed for CYP109s, whereby specific P450s’ active site cavity surface areas and volumes either increased or decreased from the open to closed confirmation, indicating their high flexibility to interact with different substrate ligands (Table 1). This increase or decrease in active site cavity surface area and volume is very common for many P450s, as this phenomenon has already been reported for bacteria CYP107 [26], CYP121 [27], and CYP102 [28].

A CYP109 member’s structural flexibility was further quantified by observing amino acid dynamics using root-mean-square difference (RMSD) (Table 2). The CYP109 members have RMSD values ranging from 0.5 Å to 2.9 Å, indicating a significant change in amino acid positions (Table 2). An interesting point to note is that CYP109E1 had both the lowest (bound to corticosterone) and highest (bound to testosterone) RMSD values from the open to closed confirmation, indicating structural flexibility and tight binding to ligands for this enzyme (Table 2). A notable point is that most CYP109 structures are from *Bacillus* species except for one CYP109, which is from *Chondromyces apiculatus* DSM 436. This indicates that the CYP109Q5 from the *C. apiculatus* structure is highly similar to *Bacillus* CYP019s, as the most changes were observed in *Bacillus* CYP109E1. The majority of CYP109 active site cavity amino acids from open to closed conformations seem to be highly conserved, indicating that these amino acids play a key role(s) in active site cavity architecture maintenance (Table 2). However, some amino acids are found uniquely to be in either the open or closed conformation, indicating their possible role in the catalysis (Table 2).

The active site cavity dynamics of CYP109E1 were the highest concerning changes in average volume compared with CYP121A1, CYP107FH5, and CYP102A1 (Table 3). However, in the case of CYP109E1, the change in the average volume was found to be increased from the open to closed confirmation, which is quite the opposite in comparison with the case of CYP107FH5 (Table 3). The change in average surface area for CYP109E1 was the lowest compared with CYP107FH5 and CYP102A1 (Table 3). Interestingly, in all three P450s, the change in average surface area was reduced from the open to the closed conformation (Table 3).

### 2.4. CYP109E1

#### 2.4.1. The Type of C17 Substituent on Steroids Determines the Catalysis

**Figure 4:**
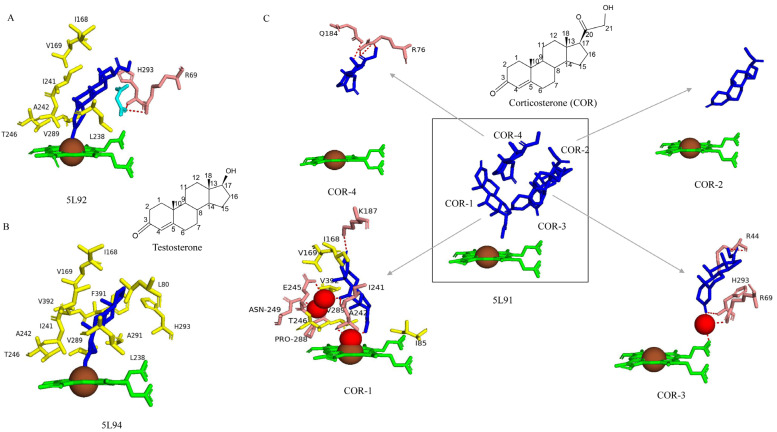
Analysis of CYP109E1 interactions with steroids such as corticosterone (COR) (**A**,**C**) and testosterone (**B**). Heme is shown in green, iron is shown as a brown sphere, steroids are shown in blue, and malonic acid is shown in cyan. Amino acids sharing van der Waals and hydrophobic interactions are shown in yellow, and amino acids sharing a direct and water-mediated interaction with the ligand are shown in salmon. Polar interactions are red dashed lines, and water molecules are red dots (4C-COR-1 and COR-3). Amino acid residues are labeled according to their single-letter codes. PDB codes are written under each modeled protein structure. Amino acids found within 5 Å of the ligands are listed in Table 4. The chemical structures of COR and testosterone are also shown in the figure.

Structure–functional analysis of CYP109E1 with steroids revealed the molecular basis for substrate specificity and stereo- and regioselectivity [13]. Steroids such as corticosterone and testosterone were found to bind in the active site cavity with different molecular orientations, with either the C3-keto oxygen (Figure 4A) or the C16 carbon and C17 substituent directed toward the P450 heme cofactor (Figure 4B) [13]. Furthermore, four corticosterone molecules were found to be bound in the active site cavity (Figure 4C), indicating the large size of the CYP109E1 active site cavity as discussed in Section 2.3. Furthermore, the CYP109E1 active site cavity was also found to accommodate steroid molecules and other molecules, such as malonic acid, indicating the dynamic nature of the active site cavity (Figure 4A). The binding of a single steroid molecule causes large structural changes such as widening of the central I helix that causes the formation of a successful water channel and/or proton delivery network for catalysis. The changes in the active site cavity surface area and volume of CYP109E1 are the highest among other CYP109s and are comparable to other P450s (Table 3), indicating enormous structural changes occur upon binding of the steroid molecule.

Analysis of CYP109E1 interactions with corticosterone and malonic acid revealed substrate-interacting amino acids (Figure 4A) (Table 4). Corticosterone is positioned roughly perpendicular to the heme plane, with its C3-keto oxygen atoms coordinating the heme-iron at the sixth axial position (Figure 4A) [13]. This steroid β-face is positioned toward the I helix, whereas the C17 substituent groups point away from the heme toward the active site pocket entrance. Amino acids Leu80, Ile168, Val169, Leu238, and Ile241 form hydrophobic interactions with corticosterone (Figure 4A) [13].

The amino acids Arg69, Ile85, Leu292, and Phe391 interacted with steroid via van der Waals interactions, and His293 interacted via hydrogen bonding with malonic acid (Figure 4A) [13]. Furthermore, our analysis revealed Arg69 can also interact through a hydrogen bond with malonic acid (Figure 4A).

Analysis of the CYP109E1 crystal structure with four bound corticosterone molecules revealed different binding orientations. One of the orientations includes corticosterone pointing toward the heme with its large C17-substituent, and its C21-hydroxyl group coordinates to the heme-iron by forming a hydrogen bond with the sixth water ligand (Figure 4C) [13]. This corticosterone interacted with residues Ile85, Ile168, Val169, Thr246, Val289, and Val392 through multiple hydrophobic contacts, as well as forming two direct hydrogen bonds with Lys187 and Ile241 and four water-mediated hydrogen bonds with Glu245, Ala242, Asn249, and Pro288 [13] (Figure 4C and Table 4). However, none of these binding orientations resulted in the catalysis of corticosterone oxidation. Thus, despite binding to CYP109E1, corticosterone is not a substrate.

Analysis of the CYP109E1–testosterone crystal structure complex revealed that testosterone is bound roughly perpendicular to the heme plane (Figure 4B) [13]. The testosterone steroid β-face is directed toward the I helix, whereas the C17 substituents point away from the heme and toward the active site pocket entry. The amino acids Leu80, Ile168, Val169, Leu238, Ile241, Ala242, Thr246, Val289, Ala291, His293, Phe391, and Val392 interact with testosterone via hydrophobic interactions (Figure 4B) [13]. Despite having similar molecular structures, corticosterone and testosterone differ at the C17 substituent, where corticosterone has a large substituent with a hydroxyl group. This suggests that while both testosterone and corticosterone can bind to CYP109E1, only testosterone undergoes catalytic conversion by this enzyme because its small C17 substituent enables 16-β hydroxylation to occur easily.

#### 2.4.2. Four Amino Acids (Val169, Lys187, Ile241, and Thr246) Are Important for Catalytic Activity

In order to identify amino acids playing a role in catalysis, a single mutation of each of the five amino acids, Val169, Lys187, Ile241, Glu245, and Thr246, to Ala was carried out [13]. The variants of V169A and I241A resulted in a complete loss of activity, and the K187A variant also had a moderate loss of catalytic activity [13]. Compared with the wild-type, the T246A variant significantly decreased CYP109E1 activity, whereas the G245A variant did not affect the production of 16β-hydroxytestosterone [13].

#### 2.4.3. Thr246 Plays a Role in the Proton Shuttle

**Figure 5:**
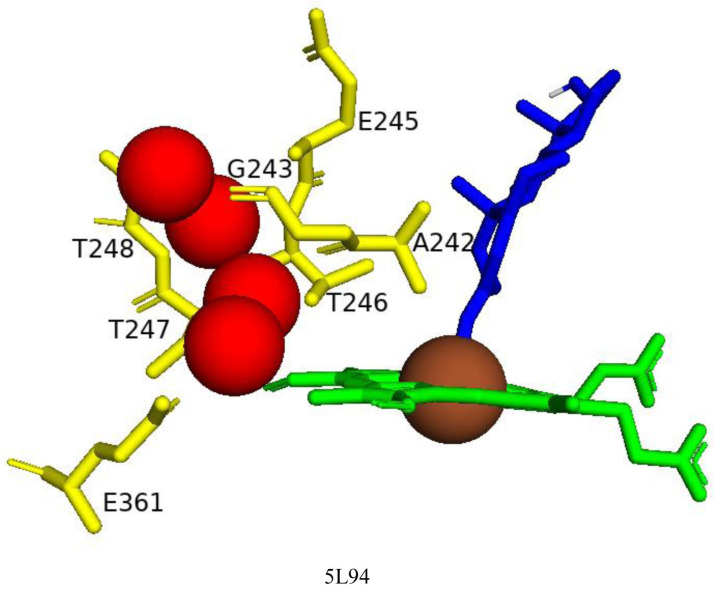
Analysis of the proton delivery network in CYP109E1 bound with testosterone. Heme is shown in green, iron is shown as a brown sphere, ligand is shown in blue, and water molecules are shown in red spheres. Amino acids within 5 Å are shown in yellow. Amino acid residues are labeled according to their single-letter codes. The PDB code is written under the modeled protein structure. Amino acids found within 5 Å of the ligands are shown in Table 4.

Analysis of the CYP109E1–testosterone crystal structure complex revealed a proton shuttle network that transfers electrons for catalysis (Figure 5) [13]. In the active site cavity, four water molecules create a continuous hydrogen-bonded network with the main-chain carbonyls of Ala242 and Gly243, the side-chain hydroxyl groups of Thr246 and Thr247, and the main-chain amide of Thr248 forming this network [13]. Amino acid mutation analysis revealed that only the T246A variant resulted in the loss of catalytic activity, and no loss of activity was observed for the G245A variant, indicating that only Thr246 plays a key role in the proton transfer required for catalysis [13]. The proton shuttle network system was also identified in other P450s, such as CYP107, where Ala245 was found to play a key role [26].

### 2.5. CYP109B4

#### 2.5.1. Asn77 Hydrophilic Interaction Is Crucial in Positioning Testosterone for C16β-Hydroxylation

**Figure 6:**
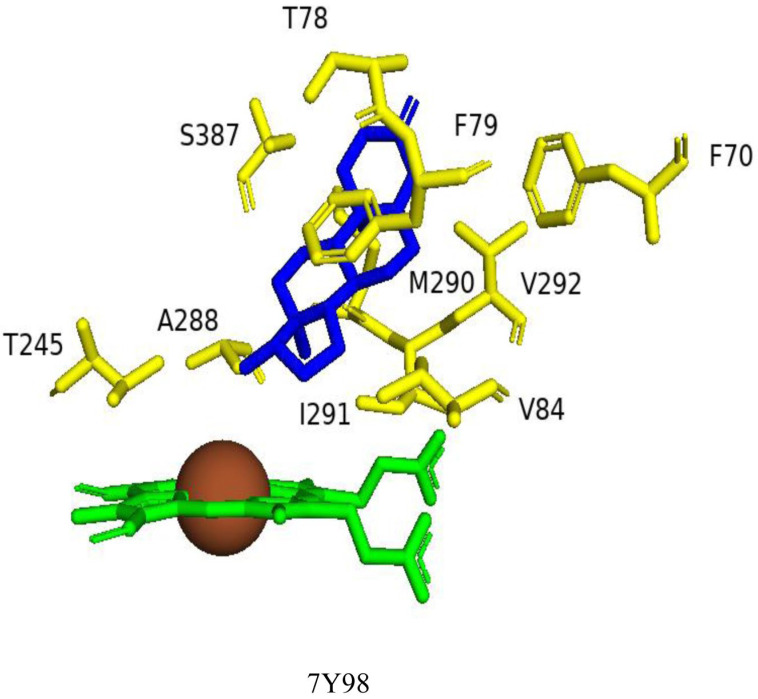
Analysis of CYP109B4 interactions with testosterone. Heme is shown in green, iron is shown as a brown sphere, and the ligand is shown in blue. Amino acids sharing hydrophobic interactions with the ligand are shown in yellow. Amino acid residues are labeled according to their single-letter codes. PDB codes are written under the modeled protein structure. Amino acids found within 5 Å of the ligands are shown in Table 4.

Structure–function analysis of CYP109B4 from *B. sonorensis* revealed the amino acids playing a key role in the proper orientation of testosterone in the binding pocket (Figure 6) such that the hydroxylation occurs at the 16β-position [19]. The hydrophobic amino acids mainly surround testosterone in the binding pocket, and interestingly, there is a hydrophilic interaction by Asn77 to the C3-keto positioned testosterone roughly perpendicular to the binding site, bringing the C16 of testosterone in close proximity to the heme iron and guiding the regioselective hydroxylation (Figure 6) [19]. The distance between the C16 of testosterone and the heme iron in CYP109B4 was the same as for CYP154C5 [19,29]. However, these two P450s show different monooxygenase stereoselectivity, whereby CYP109B4 hydroxylates at the beta-position while CYP154C5 hydroxylates at the alpha-position [19,29].

#### 2.5.2. Val84, Val292, and Ser387 Determine the CYP109B4 Regioselectivity

**Figure 7:**
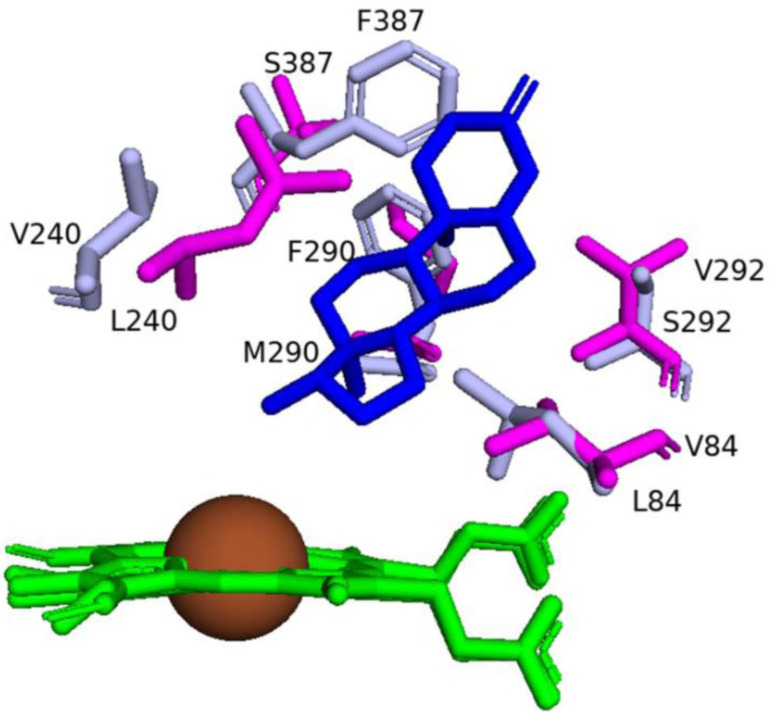
Superimposition of amino acids determining the regioselectivity of CYP109B4 (7Y98) and its variant (7Y9O). Hemes are shown in green, iron is shown as a brown sphere, and the ligand is shown in blue. The 7Y9O amino acids are shown in light blue, and the 7Y98 amino acids are shown in magenta. The ligand comes from the structure with PDB code 7Y98. Amino acid residues are labeled according to their single-letter codes.

Structure-guided mutagenesis was successfully employed in changing the regioselectivity of CYP109B4 catalysis [19]. Multiple sequence alignment and active site superimposition of CYP109B4 with different CYP109Bs, especially with CYP109B1, which hydroxylates testosterone at the 15β-position [20], identified three amino acids, Val84, Val292, and Ser387, as key for CYP109B4 regioselectivity [19]. Subsequent site-directed mutagenesis studies of these residues further confirmed their role in catalytic regioselectivity. Variants of V84I and V292R showed that the 15β-selectivity increased while the activity decreased [19]. The S387V variant exhibited a noticeable decrease in both activity and 15β-selectivity compared with wild-type CYP109B4 [19]. Extensive analysis of different variants of CYP109B4 finally resulted in the generation of a variant named B4-M7 (L240V/S387F/V84L/V292S/I291T/M290F/F294I) that completely switched regioselectivity from the 16β to 15β position (Figure 7). A schematic diagram representing the superimposition of the wild-type and variant amino acids of CYP109B4 that determine the regioselectivity is shown in Figure 7.

### 2.6. CYP109A2

#### Right Positioning and Orientation of Testosterone Toward Heme Is Essential for Catalysis

**Figure 8:**
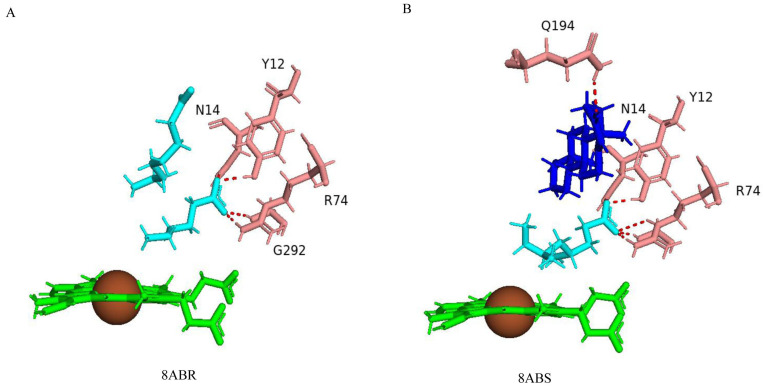
Analysis of CYP109A2 crystal structures bound with fatty acids and testosterone. (**A**) CYP109A2 interactions with hexanoic acid and octanoic acid. (**B**) CYP109A2 interactions with 4,6-dimethyloctanoic acid and testosterone. Fatty acids are shown in cyan, and testosterone is shown in blue. Heme is shown in green; iron is shown as a brown sphere. Amino acids sharing a direct polar interaction with the ligand are shown in salmon. Polar interactions are shown as red dashed lines, and amino acid residues are labeled according to their single-letter codes. PDB codes are written under each modeled protein structure. Amino acids found within 5 Å of the ligands are shown in Table 4.

To understand the structural determinant involved in directing the regio- and stereoselective hydroxylation of testosterone and vitamin D3 (VD3) by CYP109A2 of *B. megaterium* DSM319, a combined approach of X-ray crystallography and computational modeling was explored [17]. CYP109A2 hydroxylates VD3 at the C25 position but also converts testosterone into 16β-hydroxytestosterone [17]. The crystal structure of CYP109A2 was found to have bound fatty acids deriving from the *Escherichia coli* heterologous host used for CYP109A2 expression and purification, and after successful washing, the authors were able to see one bound testosterone in a non-productive position (Figure 8) [17]. However, these two crystal structures were good enough to identify the amino acids involved in the catalysis. Two fatty acid molecules were found bound in the active site cavity in one crystal structure (Figure 8A), and a single fatty acid and single testosterone molecule were found bound in another crystal structure (Figure 8B), indicating that this P450’s active site cavity is large.

The two fatty acids in the crystal structure were identified as hexanoic acid, which is located in close proximity to the heme, and octanoic acid, located a further distance away from the heme (Figure 8A) [17]. The hexanoic acid carboxylate group forms a salt bridge with the side chain of Arg74 and hydrogen bonds with the backbone amide of Gly292 and the side chains of Tyr12 and Asn14. Additionally, the acyl chain engages in hydrophobic interactions with the side chains of Ile291 and Phe389 while aligning closely with one of the heme’s propionate moieties (Figure 8A) [17]. The octanoic acid molecule is positioned toward the guanidinium group of Arg186, with its acyl chain forming hydrophobic contacts with Val240, Leu167, Val168, Gln80, and Leu84 (Figure 8A) [17].

In the second CYP109A2 crystal structure complex, a testosterone molecule occupies the ligand position far from the heme, while 4,6-dimethyloctanoic acid was modeled as the ligand near the heme (Figure 8B) [17]. The bound testosterone molecule is primarily stabilized by hydrophobic interactions [17]. These interactions occur between its carbon ring skeleton and a set of residues like those that bind the distant fatty acid, octanoic acid [17]. Additionally, testosterone C3-keto oxygen forms a hydrogen bond with the side chain of Gln194, and the C17-hydroxyl group forms water-mediated hydrogen bonds with the side chain of Arg186 and the main-chain carbonyl of Leu167 (Figure 8B) [17]. Since the crystal structures did not result in the productive position of testosterone, the molecular docking of testosterone and VD3 coupled with molecular dynamics simulation was performed to achieve the productive complex and to identify amino acids playing a key role(s) in substrate binding or catalysis. The productive CYP109A2 complex with VD3 and testosterone revealed that a specific core of amino acids keeps both steroids bound in a productive binding position, despite these steroids binding in different conformations in the enzyme active site. The core of amino acids includes six apolar and three polar/charged amino acids that stabilize the steroid binding [17]. The nine amino acids of the core are Tyr12, Asn14, Glu78, Leu84, Met85, Arg186, Leu237, Ile288, and Phe389. The majority of these residues form hydrophobic and van der Waals contacts with the steroids. Asn14, Glu78, and Arg186 form hydrophobic interactions and generate hydrogen bonds for stabilizing the C3 polar substituent of the steroids. Furthermore, Ala241 and Thr245 are also found to be important for productive binding of testosterone [17]. One surprising finding of this study is the role of Arg74. A variant of Arg74Val resulted in the loss of hydroxylation activity, indicating its importance in catalysis.

## 3. Materials and Methods

### 3.1. Genome Data Mining and Annotation of CYP109s

A single representative CYP109 protein from bacteria, CYP109A2 from *B. megaterium* (NCBI protein ID: WP_047933445.1) and two CYP109 proteins from archaea, CYP109AY1 from *Candidatus marsarchaeota* G1 archaeon OSP_C (NCBI protein ID: PSN87358.1) and CYP109BK1 from *Halocatena salina* (NCBI protein ID: WP_247995870.1) from a previous study [12], were selected as CYP109 reference proteins for genome data mining of homologous CYP109s. Initial multiple sequence alignment analysis revealed that the two archaeal CYP109s have a very low sequence percentage identity to the previously named bacterial CYP109s [12]. Subsequently, the two archaeal CYP109s were employed for the identification of homologs that may have the lowest similarity to the named CYP109s from all domains of life. Using these three reference proteins, a protein BLAST (https://blast.ncbi.nlm.nih.gov/Blast.cgi?PROGRAM=blastp&PAGE_TYPE=BlastSearch&LINK_LOC=blasthome (accessed on 25 April 2025)) was performed at the National Center for Biotechnology Information (NCBI) with a maximum of 5000 hits. The hit sequences were downloaded, and the sequences with ≥40% identity were selected. Three sets of sequences were compared, and duplicates were removed. The final set of sequences was used to perform BLAST at the P450ATLAS database [9] to identify the P450 subfamily. The proteins that showed > 55% identity were assigned to the same subfamily, and the proteins that showed less than 55% identity to the named CYP109s were assigned to new subfamilies [30].

### 3.2. Phylogenetic Analysis of the CYP109 Family

The phylogenetic tree of the CYP109 family members was constructed following the procedure previously described by our laboratory [31]. The T-REX web server has all the necessary tools to construct a phylogenetic tree [32]. At this web server, CYP109 members’ protein sequences were aligned using the MAFFT v6.8.84 program [33], and then the tree was constructed using the maximum likelihood method. Finally, the tree was visualized, colored, and generated using the web application Interactive Tree Of Life (Itol) [34].

### 3.3. Retrieving of CYP109 Crystal Structures

Twelve CYP109 crystal structures were retrieved from the Research Collaboratory for Structural Bioinformatics Protein Data Bank (RCSB PDB) [35] and used in this study. The list of CYP109s used in this study is provided in Table 1.

### 3.4. CYP109 Active Site Analysis

The individual crystal structures of the CYP109s were analyzed and classified as either in the open conformation (without any bound ligand) or in the closed conformation (with a ligand bound) [26]. The area and volume of the active site cavities in both open and closed CYP109 crystal structures were analyzed using the Computed Atlas of Surface Topography of Proteins (CASTp) version 3.0 [36]. For active site analysis, each PDB file was individually uploaded onto PyMOL software, Version 2.2.5 [37]. Using heme as the center point of the binding pocket, the active site cavities were selected including amino acid residues within 5 Å. If the bound ligand extended out of the selected binding pocket, 5 Å from the ligand was chosen instead [26]. The amino acid residues were analyzed for both open and closed conformations. As a result, conserved and unique amino acids were identified in both the open and closed conformations [27]. The active site was represented by the heme in open conformation and the heme and other ligands in closed conformation within the binding pocket [27]. The amino acid residues were represented as sticks and labeled using the one-letter amino acid codes [26].

### 3.5. Analysis of Ligand Interactions in Closed Conformation

Out of the 12 CYP109 crystal structures, 7 were in the closed conformation. Individual CYP109 PDB files were uploaded onto PyMOL, and the active site cavity was selected as described above. The amino acids were represented using stick models and labeled according to their single-letter amino acid codes [26]. Polar contacts with relevant atoms were selected. If ligand interactions with amino acid residues were present, dashed lines were shown connecting the ligand and the specific amino acid residue, water molecule, or solvent molecule [26]. If interactions between the ligand and water molecules were determined, polar interactions with the water molecules were then selected to analyze water-mediated bonds with the ligand [27]. Using published data, hydrophobic residues within 5 Å were selected and represented as sticks. The amino acid residues not interacting with the ligand were removed [26].

### 3.6. Annotation of P450 Characteristic Secondary Structures and Identification of Substrate Recognition Sites (SRSs)

P450 convention names for alpha helices and beta sheets [38] for CYP109K1-K2 was carried out using SecStrAnnotator [39], and alpha helices and beta sheets are presented in red and blue fonts. CYP109 P450s Substrate Recognition Sites (SRSs) were identified following the methods described elsewhere [38,40]. SRS1 was mapped between alpha helices B and C along the BC-loop, alpha helices F and G and their loops mapped as SRS2 and SRS3, the center portion of the alpha helix I as SRS4, N-terminus of beta sheet 1–4 as SRS5, and the beta turn at the end of the beta sheet 4 as SRS6 [38,40].

## 4. Conclusions

The steroid pharmaceutical sales market, especially concerning androgens and anabolic steroids, is expected to reach USD 78.46 billion in 2025 [41]. To meet the demand, pharmaceutical companies are investing in eco-friendly technologies that generate regio- and stereospecific hydroxylated steroids. This chemistry can be successfully achieved by using microbial enzymes, particularly cytochrome P450 monooxygenases (CYPs/P450s). P450s are well known to perform regio- and stereospecific hydroxylation of steroids, generating highly valuable pharmaceutical steroids. Research is ongoing to understand the molecular details that govern the regio- and stereoselectivity in order to enable P450 design to generate hydroxylated steroids. The present study is such an example, wherein we have undertaken a comprehensive analysis of CYP109 family members’ structure–function analysis, unraveling the factors responsible for regio- and stereospecific hydroxylation of steroids. We have performed data mining to discern the distribution of CYP109 family members across the domains of life and determined that these family members are present only in bacteria and archaea to date. Our work strongly supports the hypothesis, originally proposed by our laboratory [12], that CYP109 genes undertook horizontal gene transfer from bacteria to archaea, leading to the evolution of P450s in archaea.

**Figure 9:**
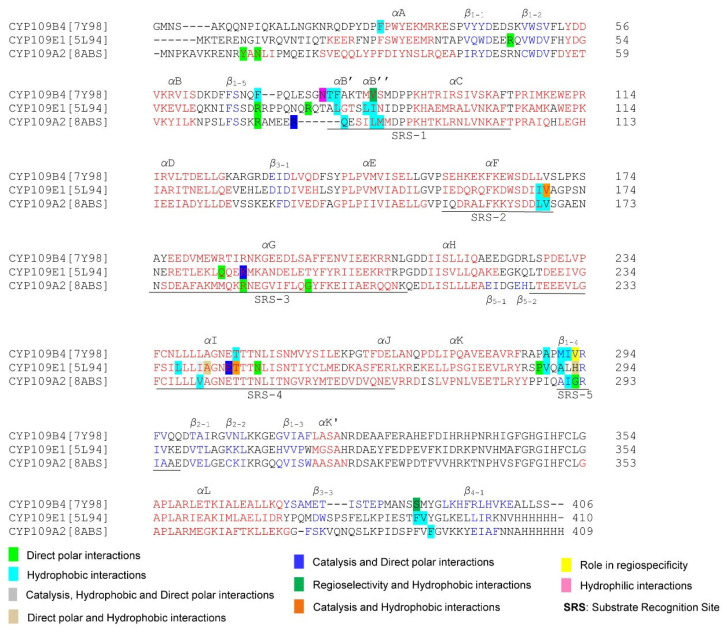
P450 characteristics and secondary structural motif analysis of selected CYP109 family members. Alpha helices and beta sheets are highlighted in red and blue. The role of various amino acids is indicated with different color backgrounds and described in the text.

The comprehensive crystal structure analysis of CYP109 members revealed amino acids that play key roles in substrate binding, orientation, and catalysis and are involved in regio- and stereospecific hydroxylation of steroids (Figure 9). This study identified that the CYP109 members’ active sites are large enough to accommodate both structurally different and multiple ligands. The RMSD value for CYP109 members revealed significant shifts in amino acid positions affecting their activity and substrate selectivity. The molecular flexibility of CYP109 members is comparable to that of CYP107 family members [26]. Amino acid residues in the active site of CYP109 members that share hydrophobic and polar interactions with substrates play vital roles in substrate binding orientation and specificity (Figure 9). The substitution of amino acids within the binding pocket of CYP109 proteins revealed significant effects on their catalytic activity and selectivity. One of the important findings revealed that sequence-guided mutational analysis resulted in the generation of a variant CYP109B4 (named B4-M7) that completely switched the oxidative regioselectivity from the 16β to the 15β position, indicating significant progress in understanding the factors governing P450 catalysis and thus allowing for P450 design for the synthesis of specific steroids. The majority of the CYP109 amino acids involved in various interactions with ligands, proven to play roles in catalysis and regio- and stereoselectivity, are found in different substrate recognition sites (SRSs) (Figure 9).

The present study provides comprehensive information on CYP109 structure–function analysis and reveals new insights regarding CYP109 active sites’ molecular dynamics, including the role of specific amino acids involved in enzymatic catalysis. The information presented herein serves as a foundation for the future genetic engineering of CYP109 enzymes to produce novel and efficacious steroids with potential medical and biotechnological applications.

## Figures and Tables

**Table 1:** Comparative analysis of CYP109 family members’ active site cavity surface area and volume.

P450 Name	Organism	PDB CODE	Surface Area (Å^2^)	Volume (Å^3^)	Conformation	Change in Surface Area (Å^2^)	Change in Volume (Å^3^)
CYP109B1	*Bacillus subtilis* subsp. *subtilis*	4RM4	1682	1652	Open	na	na
CYP109Q5	*Chondromyces apiculatus* DSM 436	6GMF	1161	769	Open	na	na
CYP109E1	*Priestia megaterium* DSM 319 (formerly *Bacillus megaterium*)	5L90	1633	2699	Open	-	-
CYP109E1	*Priestia megaterium* DSM 319	5L91	1762	2809	Closed	−129	−110
CYP109E1	*Priestia megaterium* DSM 319	5L92	2021	2260	Closed	−388	439
CYP109E1	*Priestia megaterium* DSM 319	5L94	1569	1391	Closed	64	1308
CYP109B4	*Bacillus sonorensis* L12	7Y97	1515	1793	Open	-	-
CYP109B4	*Bacillus sonorensis* L12	7Y9O	875	646	Closed	640	1147
CYP109B4	*Bacillus sonorensis* L12	7Y98	1773	1965	Closed	−258	−172
CYP109A2	*Priestia megaterium* DSM 319	5OFQ	1561	1337	Open	-	-
CYP109A2	*Priestia megaterium* DSM 319	8ABS	961	647	Closed	600	690
CYP109A2	*Priestia megaterium* DSM 319	8ABR	806	446	Closed	755	891

Note: A negative value indicates a decrease in the surface area or volume from open to closed confirmation for a specific P450 upon ligand binding. The abbreviation “na” indicates that the particular P450’s crystal structure is “not available” in both open and closed conformations, and thus changes in surface area and volume cannot be calculated. 5L91 is indicated as open confirmation in the published article [13]. However, this CYP109 has four corticosterone molecules bound in the active site, and one corticosterone interacts with heme. Considering this finding and consistency with other CYP109s analyzed in this study, this structure is considered a closed confirmation.

**Table 2:** The active site cavity dynamics analysis for CYP109 family members. The unique amino acid residues found in the active site cavities of three CYP109 members belonging to the same subfamily in open and closed conformations are presented in this table.

P450 Name	PDB Code	Conformation	Number of Amino Acids in the Active Site Cavity	Common Amino Acids	Unique Amino Acids (Found Only in Either Open or Closed Confirmation)	Amino Acids Interacting With the Ligand	RMSD (Å)
CYP109B4	7Y97	Open	34	33	Ala354	-	0.7
	7Y98	Closed	33		-	Phe70, Thr78, Phe79, Val84 *, Thr245 *, Ala288 *, Met290, Ile291 *, Ser387	
	7Y97	Open	34	30	Val84, Ile291, Val92, Phe234	-	2.4
	7Y9O	Closed	37		Ile60, Leu84, Ala286, Thr291, Ser292, Ile342, Glu360	-	
CYP109E1	5L90	Open	34	33	Gly243	-	2.7
	5L92	Closed	36		Ser67, Ala355, Glu361	**Arg69** *, Leu80, Ile168, Val169, Leu238 *, Ile241, Ala242 *, Thr246 *, Val289 *, **His293**	
	5L90	Open	34	33	Leu239	-	0.5
	5L91	Closed	35		Ser67, Glu361	**Arg44**, Ile85 *, Ile168, Val169, **Lys187**, **Ile241**, **Ala242** *, **Glu245**, **Asn249**, **Pro288**, Thr246 *, Val289 *, **His293**, Val392	
	5L90	Open	34	33	Gly243	-	2.9
	5L94	Closed	35		Ala355, Glu361	Leu80, Ile168, Val169, Leu238 *, Ile241, Ala242 *, Thr246 *, Val289 *, Ala291, His293, Phe391, Val392	
CYP109A2	5OFQ	Open	33	33	-	-	2.5
	8ABR	Closed	40		Ser72, Arg74, Leu149, Asn243, Phe346, Ala354, Glu360	**Tyr12**, **Asn14**, **Arg74** *, Gln80, Leu84 *, Leu167, Leu168, Val240, Ile291 *, **Gly292** *, Phe389	
CYP109A2	5OFQ	Open	33	33	-	-	2.5
	8ABS	Closed	40		Ser72, Arg74, Leu149, Asn243, Phe346, Ala354, Glu360	**Tys12**, **Asn14**, **Arg74** *, Gln80, Leu84 *, **Leu167**, Val168, **Arg186**, **Gln194**, Val240	

Note: Amino acids in bold represent direct polar contacts, and amino acids underlined represent hydrophobic contacts. The symbol * indicates amino acids that interact with the substrate and are also found in the active site cavity of an enzyme.

**Table 3:** Comparative analysis of the surface area, volume, and root-mean-square deviation (RMSD) between CYP109E1 and other P450s.

P450 Name	Change in Average Area (Å^2^)	Change in Average Volume (Å^3^)	RMSD (Å)	Reference
CYP107FH5	276 ^#^	494 ^#^	3.0	[26]
CYP121A1	37	8	0.2	[27]
CYP102A1	179 ^#^	23 ^#^	4.4	[28]
CYP109E1	151 ^#^	545	2.9	Current work

Symbol: #, the active site surface area and volume decreased from open to closed conformation. The highest change in the average surface area and volume for the respective P450 is presented.

**Table 4:** List of amino acid residues surrounding the ligand within 5 Å in CYP109 P450s.

PDB CODE	Amino Acid Residues
5L91 COR-1	Ile85, Ile168, Val169, Ala170, **Lys187**, **Ile241**, **Ala242**, **Glu245**, **Asn249**, **Pro288**, Thr246, Val289, Ala291, Leu292, Ser389, Phe391, Val392
5L91 COR-2	Arg69, Pro71, Gln75, Leu80, Gly81, Ser83, Ile85, Asn86, Leu238, Ile241, Ala242
5L91 COR-3	**Arg44**, Val46, **Arg69**, Ala291, Leu292, **His293**, Arg294, Phe391, His312
5L91 COR-4	**Arg76**, Thr78, Leu80, Ile168, **Gln184**, Lys187, Met188, Asn191, Ile241
5L92	**Arg69**, Leu80, Ile85, Asn86, Ile168, Val169, Leu238, Ile241, Ala242, Thr246, Val289, **His293**, Cys352, Phe391, Val392
5L94	Leu80, Ile85, Ile168, Val169, Leu238, Ile241, Ala242, Glu245, Thr246, Val289, Ala291, Leu292, His293, Cys352, Ser389, Phe391, Val392
7Y98	Phe70, N77, Thr78, Phe79, Val84, Ala241, Thr245, Ala288, Met290, Ile291, Val292, Ser387
7Y9O	Leu84, Leu237, Ala241, Thr245, Ala288, Cys351
8ABS	**Tys12**, **Asn14**, **Arg74**, Glu77, Gln80, Glu81, Ser82, Leu84, Met85, Val168, **Leu167**, **Arg186**, Val190, **Gln194**, Gly233, Phe234, Ile236, Leu237, Val240, Ala241, Glu244, Thr245, Ile288, Ala290, Ile291, Gly292, Arg293, Phe389
8ABR	**Tyr12**, **Asn14**, **Arg74**, Glu78, Gln80, Ser82, Leu84, Met85, Leu167, Leu168, Arg186, Val190, Ile236, Leu237, Val240, Ala241, Glu244, Thr245, Ile288, Ala290, Ile291, **Gly292**, Arg293, Phe389

Note: Amino acids in bold represent direct polar contacts, and amino acids underlined represent hydrophobic contacts.

## Data Availability

Data are contained within the article.

## Supplementary Materials

The following supporting information can be downloaded at: https://www.mdpi.com/article/10.3390/ijms26136219/s1.
